# Reactive oxygen species: role in the development of cancer and various chronic conditions

**DOI:** 10.1186/1477-3163-5-14

**Published:** 2006-05-11

**Authors:** Gulam Waris, Haseeb Ahsan

**Affiliations:** 1 Moores UCSD Cancer Center, University of California at San Diego, La Jolla, CA 92093, USA; 2 Department of Dermatology, University of Wisconsin – Madison, Medical Science Center, Madison, WI 53706, USA

## Abstract

Oxygen derived species such as superoxide radical, hydrogen peroxide, singlet oxygen and hydroxyl radical are well known to be cytotoxic and have been implicated in the etiology of a wide array of human diseases, including cancer. Various carcinogens may also partly exert their effect by generating reactive oxygen species (ROS) during their metabolism. Oxidative damage to cellular DNA can lead to mutations and may, therefore, play an important role in the initiation and progression of multistage carcinogenesis. The changes in DNA such as base modification, rearrangement of DNA sequence, miscoding of DNA lesion, gene duplication and the activation of oncogenes may be involved in the initiation of various cancers. Elevated levels of ROS and down regulation of ROS scavengers and antioxidant enzymes are associated with various human diseases including various cancers. ROS are also implicated in diabtes and neurodegenerative diseases. ROS influences central cellular processes such as proliferation a, apoptosis, senescence which are implicated in the development of cancer. Understanding the role of ROS as key mediators in signaling cascades may provide various opportunities for pharmacological intervention.

## 

The term cancer refers to more than hundred types of the disease. Almost every tissue in the body can spawn malignancies and some can yield several types. Cancer cells possess an even more insidious property to migrate from the site where they originate and form masses at distinct sites in the body. Cancer progression is a stepwise process where the initiated cells, nodules, polyp or the papilloma evolve further and become progressively more malignant. The genes implicated in malignancy are often modified forms of human genes. The activation of protooncogenes into oncogenes may contribute to malignancy. Mutations can also convert protooncogenes into carcinogenic oncogenes [1,2].

## Reactive oxygen species

Reactive oxygen species (ROS) are derived from the metabolism of molecular oxygen [3]. ROS include superoxide anion radical (O_2_^-.^), singlet oxygen (^1^O_2_), hydrogen peroxide (H_2_O_2_), and the highly reactive hydroxyl radical (^.^OH). The deleterious effects of oxygen are said to result from its metabolic reduction to these highly reactive and toxic species [4].

ROS normally exist in all aerobic cells in balance with biochemical antioxidants. Oxidative stress occurs when this critical balance is disrupted because of excess ROS, antioxidants depletion, or both. To counteract the oxidant effects and to restore redox balance, cells must reset important homeostatic parameters. ROS are not always harmful metabolic byproducts; when tightly regulated, ROS can act as intracellular signaling molecules [5,6].

**Figure 1:**
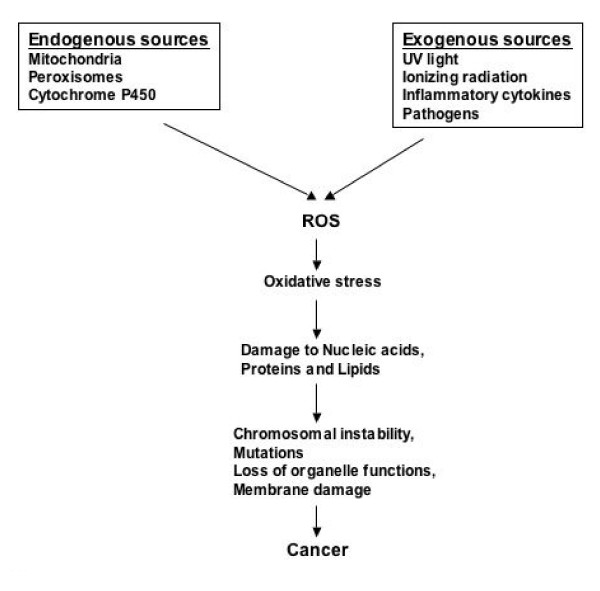
Pathways illustrating the sources of reactive oxygen species and its role in the development of cancer.

In living cells, the major source of endogenous ROS are hydrogen peroxide and superoxide anion, which are generated as by products of cellular metabolism such as mitochondrial respiration [7]. Alternatively, hydrogen peroxide may be converted into water by the enzymes catalase or glutathione peroxidase. Variability or inductive changes in the expression of these enzymes can significantly influence cellular redox potential. ROS can cause tissue damage by reacting with lipids in cellular membranes, nucleotides in DNA [8], sulphydryl groups in proteins [9] and cross-linking/fragmentation of ribonucleoproteins [10] (see figure 1). The relatively unreactive superoxide anion radical is converted by superoxide dismutase (SOD) into H_2_O_2_, which in turn take part in the "Fenton reaction", with transition metal ion (copper or iron) as catalysts, to produce the very reactive hydroxyl radical [11-14].

## Oxidative DNA damage and cancer

Damage to DNA by ROS has been widely accepted as a major cause of cancer [15]. In patients with diseases associated with a risk of cancer indicates an increased rate of oxidative DNA damage or in some instances deficient repair system such as Fanconi anemia, chronic hepatitis, cystic fibrosis and various autoimmune diseases [16-20]. Human studies support the experimentally based notion of oxidative DNA damage as an important mutagenic and apparently carcinogenic factor [21]. ROS can damage DNA and the division of cells with unpaired or misrepaired damage leads to mutations. The majority of mutations induced by ROS appear to involve modification of guanine, causing G→T transversions [22-25]. If it relates to critical genes such as oncogenes or tumor suppressor genes, initiation/progression can result [26]. Indeed, these species can act at several steps in multistage carcinogenesis. It is now assumed that ROS are involved both in the initiation and progression of cancer [27].

Mutations caused by oxidative DNA damage include a range of specifically oxidized purines and pyrimidines, alkali labile sites, single strand breaks and instability formed directly or by repair processes [28-32]. Because of the multiplicity of DNA modifications produced by ROS, it has been difficult to establish the frequency and specificity of mutations by individual oxygen radical induced lesions. Some of these modified bases have been found to possess mutagenic properties. Therefore, if not repaired they can lead to carcinogenesis. Studies show that although all the four bases are modified by ROS, mutations are usually related to modification of GC base pairs, while that of AT base pair rarely leads to mutations [33]. These mutations are usually base pair substitutions, whereas base deletions and insertions are less frequent. In human tumors, G to T transversions are the most frequent mutations in the p53 suppressor gene [34-36]. Using single stranded DNA template in a sensitive forward mutation system, various mutations, including tandem double CC→TT substitution have been observed in DNA treated with oxygen free radicals [37]. Elevated levels of modified bases in cancerous tissue may be due to the production of large amount of H_2_O_2_, which has found to be characteristic of human tumor cells [38,39]. Initiation of cancer in humans by ROS is further supported by the presence of oxidative DNA modifications in cancer tissue [26,40].

Cigarette smoke, which is rich in carcinogens such as nitrosamines and polycyclic aromatic hydrocarbons [41-44], causes accumulation of 8-hydroxydeoxyguanosine (8-OHdG). Lungs from cigarette smokers contain two to three fold higher 8-OHdG [45], that could lead to mutations, some of which might be induced by oxygen free radicals, resulting in inflammatory responses, fibrosis and tumor development [46]. Urine obtained from smokers also has a four to ten fold elevation in altered nucleotides that are known to be produced by ROS [47]. Urinary 8-OHdG is a biomarker of oxidative stress, cancer, atherosclerosis and diabetes [48].

Oxidative DNA damage may be involved in the development of breast cancer. Increased steady-state levels of DNA base damage with a pattern characteristic of ^.^OH attack have been reported in inflammatory breast disease [49] where malignant progression can occur. It is reported that elevated levels of 8-oxo-dG adducts in DNA play a fundamental role in breast cancer [50]. Evidence also exists for the progression of breast tumor to the metastatic state and is an important etiologic factor [51]. Carcinoma of hepatic cells is often associated with chronic infection by hepatitis B or C viruses or ingestion of aflatoxins [52-56]. Oxidative stress induced by these viruses represents one of the intracellular events that cause the genesis of hepatocellular carcinoma [17,57]. G→T transition has been shown to be one of the more common types of mutation produced by aflatoxin lesion and ROS damage to DNA [58]. 8-OHdG has also been reported to accumulate in hepatocellular carcinoma [59,60]. The measurement of DNA damage and mutation in human liver as a function of persistence of chronic hepatitis might be predictive for the onset of liver cancer. Chronic prostate hypertrophy is diagnosed in most males by the age of 40 yr. But the late appearance of prostatic carcinoma suggests that a multistep process is involved in tumorigenesis. The paucity of known chemical agents associated with prostate cancer indicates an association with endogenous cellular processes [61-63]. The most reasonable candidates for endogenously formed genotoxins that accumulate in later life are the ROS.

The epidemiological studies involving measurement of typical modified DNA bases in a large variety of individual tumor tissue and their respective normal tissues may provide insights into the mechanism of carcinogenesis related to ROS. Measurement of purine and pyrimidine derived DNA lesions in tissues may prove to be useful in determining an association between free radical producing agents and cancer risk.

## ROS and diseases

There is growing awareness that oxidative stress plays a role in various clinical conditions such as malignant diseases, diabetes, atherosclerosis, chronic inflammation, viral infection, and ischemia-reperfusion injury [64-69]. ROS can cause oxidative DNA and protein damage, damage to tumor suppressor genes and enhanced expression of proto-oncogenes [70-72] and oxidative stress has been shown to induce malignant transformation of cells in culture [73]. Diseases associated with oxidative stress such as diabetes mellitus and cancer show a pro-oxidative shift in the redox state and impaired glucose clearance suggesting that muscle mitochondria is the major site of elevated ROS production. This condition may be referred to as 'mitochondrial oxidative stress'. Cancer patients commonly have decreased glucose clearance capacity, high glycolytic activity and lactate production. It is, therefore, suggested that the observed pro-oxidative shift is mediated by an increased availability of mitochondrial energy substrate. The 'inflammatory oxidative conditions' are typically associated with an excessive stimulation of NAD(P)H oxidase by cytokines and other factors. The increased ROS production or changes in intracellular glutathione levels are often involved with pathological changes indicative of a dysregulation of signal cascades or gene expression [74].

ROS are potential carcinogens because they facilitate mutagenesis, tumor promotion and progression. The growth promoting effects of ROS are related to redox-responsive cell signaling cascades. Sometimes, even normal cells show increased proliferation and expression of growth-related genes if exposed to H_2_O_2 _or O_2_^-.^. Certain types of cancer cells also produce significant amounts of ROS. ROS production is induced after the expression of several genes associated with a transformed phenotype including H-Ras or mox1.

Because of its high metabolic rate and relatively reduced capacity for cellular regeneration, the brain is believed to be particularly susceptible to the damaging effects of ROS. In neurodegenerative diseases like Parkinson's, Alzheimer's and amyotrohic lateral sclerosis (ALS), ROS damage has been reported within the specific brain region that undergo selective neurodegeneration. Protein oxidation has been reported in the hippocampus and neocortex of patients with Alzheimer's disease, Lewy bodies in Parkinson's disease and within the motor neurons in ALS [75]. Lipid peroxidation has also been identified in the cortex and hippocampus of patients with Alzheimer's disease, substantia nigra of patients with Parkinson's disease and spinal fluid in patients with ALS. It is known that ROS can cause neuron and astrocyte death through apoptosis and necrosis. Mitochondria are involved in excitotoxic nerve cell death through calcium-related bursts of ROS production and opening of permeability transition pores. Oxidative stress is also related to glutamate release and NMDA receptor activation during cerebral ischemia-reperfusion, production of O_2_^-. ^in neurons and brain macrophages and glutamine-induced ROS production in astrocytes. Evidence implicating ROS in major degenerative diseases is also consistent with their role in brain aging. There is a general agreement that oxidative stress contributes to dopaminergic cell degeneration in Parkinson's disease. Oxidative stress has also been implicated as one of the earliest events in Alzheimer's disease [76].

## ROS and viral infection

Reactive oxygen metabolites play a complex role in many diseases and metabolic regulation. Because viruses replicate in living cells, such metabolites influence the growth of viruses in addition to serving as a host defense mechanism. Humans infected with viruses (HIV, hepatitis, and influenza) induce activation of phagocytes, which is associated with production of ROS. The activated phagocytes may also release pro-oxidant cytokines such as tumor necrosis factor (TNF) and interleukin-1 [77-79].

**Figure 2:**
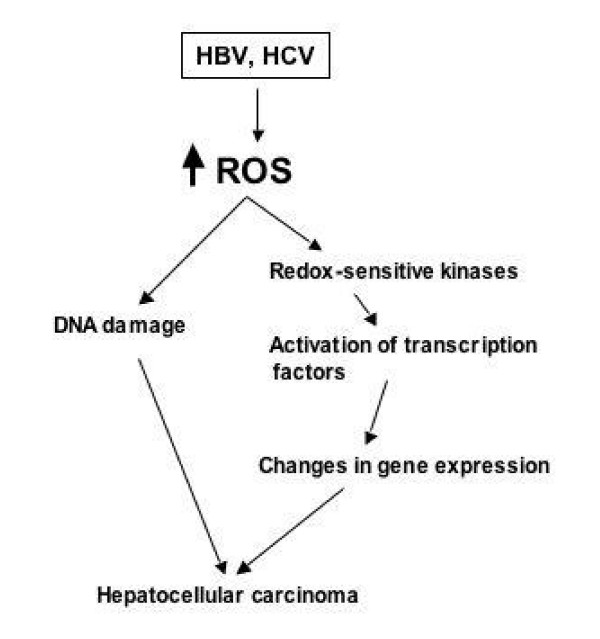
A link between hepatitis viruses and hepatocellular carcinoma. Viral gene expression regulates the cellular gene expression via oxidative stress, followed by activation of cellular kinases and transcription factors which leads to the genesis of hepatocellular carcinoma.

Chronic hepatitis B (HBV) and hepatitis C virus (HCV) infections are associated with an increased production of ROS within the liver that is responsible for the oxidation of intracellular macromolecules. Infection with these viruses can also affect the host cell pro-/antioxidant balance by increasing cellular pro-oxidants such as iron and nitric oxide and also by inhibiting the synthesis of antioxidant enzymes. Antioxidants, together with agents interfering with the harmful effects of cytokines and lipid mediators, may have a role in the treatment of viral diseases. ROS may facilitate or even promote replication of many viruses, depending on the cell and type of virus involved. Enhanced oxidative stress modulates the HCV RNA replication and hepatic cell survival via activation of oncogenic transcription factors that leads to the generation of hepatocellular carcinoma (see figure 2) [80-82]. Redox-sensitive kinases, Src, JAK, PI3K-Akt and MAPK (Erk, JNK, p38) regulate transcription factors through phosphorylation of the protein modules (see figure 2). Chronic HBV infection results in an increased total intra-hepatic iron and/or increase in the pro-oxidant low-molecular weight iron compartment of the liver. Previously, a strong correlation between the presence of HBV surface antigen and iron deposition in the Kupffer cells and spleens of infected individuals has been reported [83]. In addition to increased intracellular iron, elevated TNF-α has been found in hepatocytes from patients chronically infected with HBV [84].

Humans infected with HIV have been shown to be under chronic oxidative stress. HIV-seropositive humans exhibit decreased concentrations of naturally occurring antioxidant reductants such as total acid-soluble thiols, cysteine, and glutathione in plasma, peripheral blood monocytes, and lung epithelial-lining fluids [85]. In addition, elevated levels of hydroperoxides and malondialdehyde are found in plasma of HIV-infected individuals. In cell culture system, ROS promotes replication of HIV, and antioxidants such as NAC inhibit the replication of the virus.

Oxidative stress has been reported to affect the cellular protein kinase/phosphatase balance, which is described in a number of tumors. The exogenous oxygen radical load is contributed by a variety of environmental agents (inhaled smoke and polluted air) and dietary antioxidants [86-88]. Mutagens, tumor promoters and a variety of carcinogens including benzene, aflatoxin and benzo(a)pyrene may exert their partly by generating ROS during their metabolism [89-91].

## ROS and signaling cascades

ROS is produced in non-phagocytic cells as a result of various signaling pathways such as receptor tyrosine kinases (RTKs) which become activated by growth factors – epidermal growth factor, platelet derived growth factor, fibroblast growth factor as well as cytokines (tumor necrosis factor, γ-interferon and interleukins) leading to an intracellular tyrosine phosphorylation cascade [64]. The ROS activated signal transduction pathways are regulated by two distinct protein families – the Mitogen Activated Protein Kinase (MAPK) and the redox sensitive kinases. The MAPKs transduce signals from the cell membrane to the nucleus in response to a wide range of stimuli. MAPKs are serine/threonine kinases that, upon stimulation, phosphorylate their specific substrates at serine and/or threonine residues. Such phosphorylation events can either positively or negatively regulate substrate, and thus entire signaling cascade activity. Thus, the MAPK signaling pathways modulate gene expression, mitosis, proliferation, motility, metabolism, and programmed cell death. Conventional MAPKs consist of three family members: the extracellular signal-regulated kinase (ERK, subdivided into ERK1 and 2); the c-Jun NH2-terminal kinase (JNK, subdivided into JNK1, 2 and 3); and the p38 MAPK (subdivided into α, β, γ, and δ p38-MAPK [92].

MAPKs regulate processes important in carcinogenesis including proliferation, differentiation, and apoptosis. MAPK modulate gene expression through phosphorylation of a wide array of transcription factors. Of the three subfamilies, the ERK pathway has most commonly been associated with the regulation of cell proliferation. Activation of the ERK, JNK, and p38 subfamilies has been observed in response to changes in the cellular redox balance. The balance between ERK and JNK activation is a key determinant for cell survival as both a decrease in ERK and an increase in JNK is required for the induction of apoptosis. Activation of MAPKs directly leads to increased AP-1 activity resulting in increased cell proliferation. One of the genes regulated by AP-1 is cyclin D1. AP-1 binding sites have been identified in the cyclin D1 promoter and AP-1 activates this promoter, resulting in activation of cyclin-dependent kinase (cdks), which promotes entry into the cell division cycle. c-Jun also stimulates the progression into the cell cycle both by induction of cyclin D1 and suppression of p21^waf^, a protein that inhibits cell cycle progression. JunB, considered a negative regulator of c-jun-induced cell proliferation, represses c-jun-induced cyclin D1 activation by the transcription of p16^INK4a^, a protein that inhibits the G1 to S phase transition.

NF-κB activation has been linked to the carcinogenesis process because of its roles in inflammation, differentiation and cell growth. NF-κB regulates several genes involved in cell transformation, proliferation, and angiogenesis. Carcinogens and tumor promoters including UV radiation, phorbol esters, asbestos, alcohol, and benzo(a)pyrene are among the external stimuli that activate NF-κB. The expression of several genes regulated by NF-κB (bcl-2, bcl-x_*L*_, TRAF1, TRAF2, SOD, and A20) promotes cell survival at least in part through inhibition of apoptotic pathways. Expression of NF-κB has been shown to promote cell proliferation, whereas inhibition of NF-κB activation blocks cell proliferation. Additionally, tumor cells from blood neoplasms, and colon, breast, pancreas, and squamous cell carcinoma cell lines have all been reported to constitutively express activated NF-κB [93].

**Figure 3:**
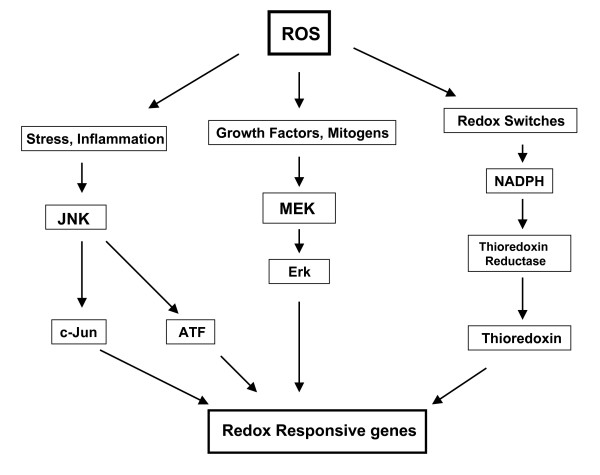
The various signaling cascades of ROS involved in the regulation and activation of redox sensitive genes.

The second family consists of signaling factors that use cysteine motifs as redox-sensitive sulphydryl switches to modulate specific signal transduction cascades regulating downstream proteins. The redox-sensitive signaling cascade involves the cytoplasmic factors (thioredoxins), nuclear signaling factors such as Ref-1 (Redox factor-1) and transcription factors (AP-1, NF-κB, Nfr-1, Egr-1). The cytoplasmic sulphydryl containing proteins such as thioredoxins are critical upstream signaling proteins that regulate multiple intracellular processes such as DNA synthesis, cell growth, etc. The signaling cascades elicited by ROS culminates in the activation of c-Jun and c-Fos subunits of the active nuclear transcription factor, AP-1 (activator protein-1), that activate genes involved in cellular proliferation. Redox-sensitive signaling factors regulate multiple processes including proliferation, cell cycle and anti-apoptotic signaling pathways. Inhibition of thioredoxins inhibits several pro-survival transcription factors such as Egr-1, AP-1 and NF-κB resulting in a G1 phase arrest [94] (see figure 3).

The role of reactive oxygen species in cell growth regulation is complex, being cell specific and dependent upon the form of the oxidant as well as the concentration of the particular reactive oxygen species. The modification of gene expression by reactive oxygen species has direct effects on cell proliferation and apoptosis through the activation of transcription factors including MAPK, AP-1, and NF-κB pathways. Oxidant-mediated AP-1 activation results in enhanced expression of cyclin D1 and cdks, which in turn promotes entry into mitosis and cell division. Likewise, reactive oxygen species function as second messengers involved in activation of NF-κB by tumor necrosis factor and cytokines. DNA damage, mutation, and altered gene expression are all required participants in the process of carcinogenesis. Although these events may be derived by different mechanisms, a common theme is the involvement of reactive oxygen species and oxidative stress in neoplastic transformation.
